# Role of the Neutrophil-Lymphocyte Ratio in the Differential Diagnosis of Exudative Pleural Effusion

**DOI:** 10.6061/clinics/2016(10)10

**Published:** 2016-10

**Authors:** Ulku Aka Akturk, Dilek Ernam, Makbule Ozlem Akbay, Nagihan Durmus Koçak, Erhan Ogur, Ilim Irmak

**Affiliations:** Sureyyapaşa Chest Disease and Thoracic Surgery Education and Research Hospital, Istanbul, Turkey

**Keywords:** Exudative Pleural Effusion, Neutrophil-Lymphocyte Ratio

## Abstract

**OBJECTIVES:**

Pleural effusion is a common diagnostic and clinical problem. The differential diagnosis of pleural effusion may be difficult and may require several procedures, including invasive ones. Certain studies have investigated biochemical parameters to facilitate the diagnosis of exudative pleural effusion; however, it remains a challenging problem in clinical practice. We aimed to investigate the potential role of the neutrophil-lymphocyte ratio, which can be easily obtained by determining the cell count of the pleural fluid, in the differential diagnosis of exudative pleural effusion.

**METHODS:**

Records from patients who underwent thoracentesis and pleural fluid analysis between May 1, 2013, and March 1, 2015, were obtained from the electronic database of our hospital. The patients who met the inclusion criteria were divided into five groups according to their diagnosis: malignant pleural effusion, para-malignant pleural effusion, para-pneumonic effusion, tuberculosis-related effusion or other. The neutrophil-lymphocyte ratio value was calculated by dividing the absolute neutrophil count by the absolute lymphocyte count. The patient groups were compared according to the given parameter.

**RESULTS:**

A total of 465 patients who met the inclusion criteria among 1616 patients with exudative pleural effusion were included in the study. The mean neutrophil-lymphocyte ratio value was significantly lower in tuberculosis-related pleural effusion compared to malignant, para-pneumonic and para-malignant effusions (*p*=0.001, *p*=0.001, *p*=0.012, respectively). The areas under the curve for tuberculosis pleurisy compared to malignant, para-pneumonic and para-malignant effusions were 0.38, 0.36, and 0.37, respectively. Lower cut-off values had higher sensitivity but lower specificity for tuberculosis pleurisy, while higher cut-off values had higher specificity but lower sensitivity for this condition.

**CONCLUSION:**

The pleural fluid neutrophil-lymphocyte ratio, which is an inexpensive, reproducible, and easily calculated hematological parameter, may facilitate the differential diagnosis of pleural effusion.

## INTRODUCTION

Pleural effusion is a common clinical problem, and its differential diagnosis may sometimes be difficult and may require several procedures, including invasive ones. The most common causes of exudative pleural effusion include cancer and tuberculosis (TB). Although many biochemical parameters, such as lactate dehydrogenase (LDH), C-reactive protein (CRP), adenosine deaminase (ADA), interferon gamma and procalcitonin levels, have been studied in the context of the diagnosis of exudative pleural effusion 1-5, its diagnosis is still challenging.

During the last few years, the neutrophil-lymphocyte ratio (NLR) has been investigated as a new inflammatory marker. Although the relationship between the NLR and lung cancer has been most frequently investigated among studies on lung diseases 6,7, a few studies have investigated use of the NLR values in the pleural fluid for the differential diagnosis of bacterial pneumonia and TB pleural effusion 8,9.

In the present study, we aimed to investigate the potential role of the NLR, which can be easily obtained by determining the cell count of the pleural fluid, in the differential diagnosis of exudative pleural effusion.

## MATERIALS AND METHODS

### Study population

This study was designed as a retrospective cohort study and conducted as a single-center study at a tertiary care hospital with a high patient bed capacity.

**Figure 1:**
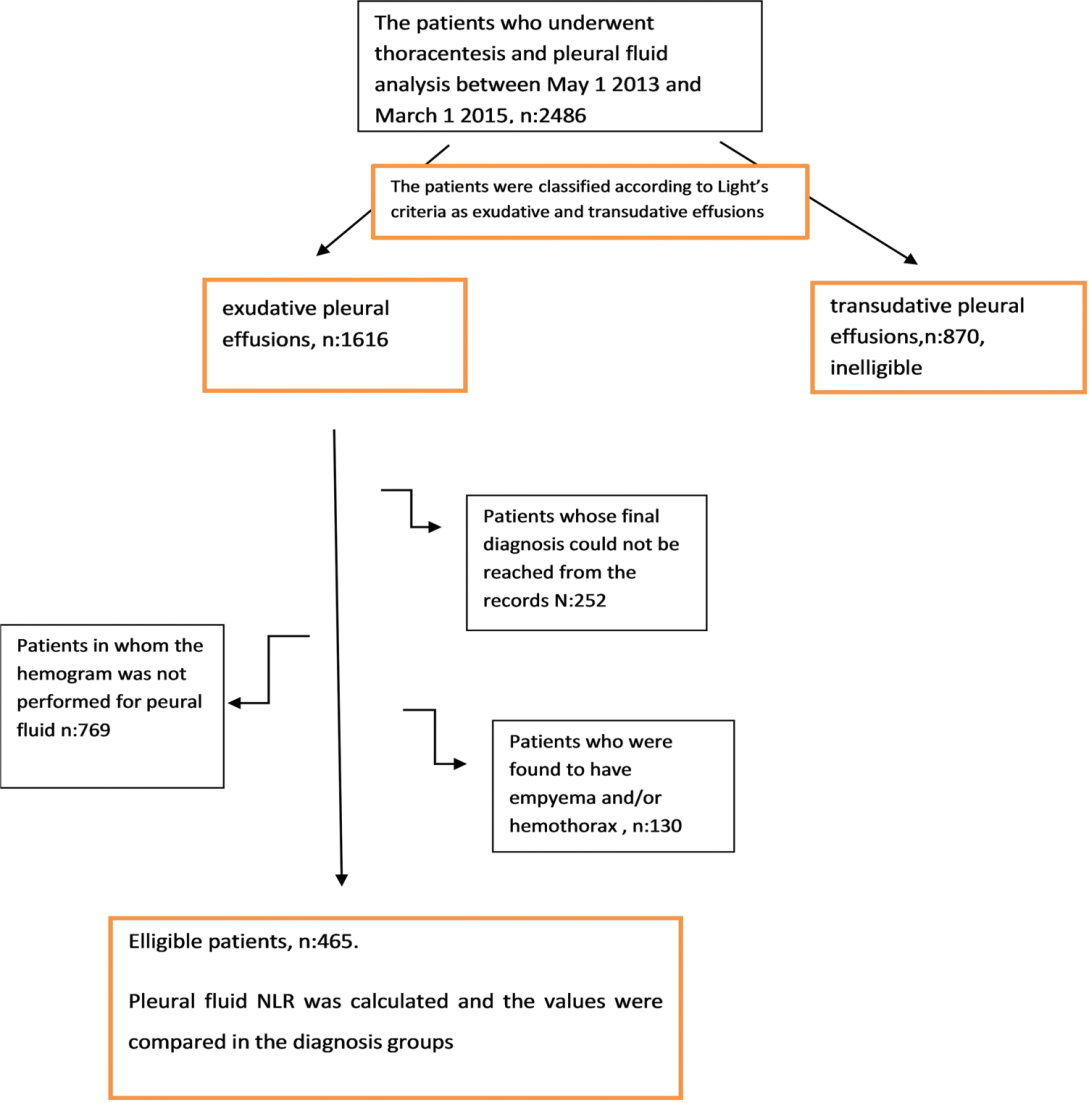
Flow chart.

The records of patients who underwent thoracentesis and pleural fluid analysis between May 1, 2013, and March 1, 2015, were obtained from the electronic database of our hospital. Patients with transudative pleural effusion according to the Light criteria 10, with empyema and/or hemothorax, whose pleural fluid cell count was not analyzed or who did not have a final diagnosis were excluded from the study (Figure 1).

The study protocol was approved by the local ethics committee of the hospital in accordance with the Declaration of Helsinki. Due to the retrospective nature of the study design, informed consent was not required.

### Data

The demographic characteristics, the final diagnoses based on clinical evaluation and invasive procedures, and the pleural fluid parameters of the patients were recorded (Figure 1). The patients were divided into five groups according to their final diagnosis: malignant pleural effusion, para-malignant pleural effusion, para-pneumonic effusion, TB pleurisy or other (e.g., chronic renal failure or rheumatic disease). In certain patients with chronic renal failure, exudative pleural effusion developed because of previously established pleural effusion. All disease groups were compared according to their NLR values.

### Definitions

The NLR value was calculated by dividing the absolute neutrophil count by the absolute lymphocyte count. These counts were obtained from a pleural fluid analysis.

Malignant pleural effusion was diagnosed when the pleural fluid cytology and/or pleural biopsy findings were positive for malignancy.

Para-malignant effusion was defined as effusion secondary to lung cancer without evidence of pleural invasion.

TB pleurisy was diagnosed according to positive mycobacterium TB culture findings for the pleural fluid and/or the presence of caseous granulomas in a pleural biopsy specimen after exclusion of other granulomatous disease.

Para-pneumonic effusion was diagnosed according to the presence of cough, fever and a radiographic pulmonary infiltrate that disappeared with antibiotics.

Renal failure was identified according to increased urea and creatinine levels and clinical evidence of both fluid overload and an absence of purulent sputum, malignancy and pulmonary infiltrates.

### Statistical analyses

The definitive statistical data are expressed as frequencies, percentages, means, standard deviations, medians, and/or quartiles. The chi-square test was used to compare categorical variables. The Mann-Whitney U test was used to compare continuous variables between two groups. A *p*-value of <0.05 was considered significant. Receiver operating characteristic (ROC) curves were also used to evaluate the diagnostic value of NLR values for predicting the diagnosis of malignant effusion, TB pleurisy, para-pneumonic effusion and para-malignant pleural effusion.

The statistical analyses were performed using the SPSS program (SPSS version 16; SPSS Inc., Chicago, IL, USA).

## RESULTS

Records from 2486 patients who underwent thoracentesis and pleural fluid analysis between May 1, 2013, and March 1, 2015, were collected from the automated database system of our hospital. A total of 465 patients who met the inclusion criteria among 1616 patients with exudative pleural effusion were included in the study (Figure 1). The mean age of the patients was 64±18 years (62.3% male; 37.5% female). According to pleural fluid analyses, pleural effusion resulted from malignant diseases in 177 (38.1%) patients and from benign diseases in 288 (61.9%) patients. The cause of the presence of pleural fluid was malignant pleural effusion in 177 (38.1%) patients, TB pleurisy in 101 (21.5%) patients, para-pneumonic pleural effusion in 91 (19.4%) patients, para-malignant pleural effusion in 45 (9.8%) and other causes (such as chronic renal failure or rheumatic disease) in 56 (11.2%) patients. The mean and median NLR values were calculated for each disease group and were compared between paired groups (Table 1).

The difference in median NLR values between malignant (;3.5, 1.6-7.5) and benign (3.3, 1.2-7.0) pleural effusions was not statistically significant (*p*=0.55). In contrast, the median NLR value was significantly lower in TB pleurisy (2.2) compared to malignant (3.5), para-pneumonic (4.1) and para-malignant (4.2) pleural effusions (*p*=0.001, *p*=0.001, and *p*=0.012, respectively). However, there was no difference between malignant, para-pneumonic and para-malignant effusions.

In view of the significantly higher values of the pleural NLR in malignant, para-pneumonic and para-malignant effusions compared to TB pleurisy, ROC analysis and determination of the cut-off values, sensitivity and specificity of this parameter for the differentiation of TB pleurisy from these effusions were carried out using paired groups. In particular, ROC curves were drawn for the NLRs of each pair of diseases, and the values of the area under the curve (AUC) were calculated (Figures 2 A, B, C , Table 2).

The AUC values for TB pleurisy compared to malignant, para-pneumonic and para-malignant effusions were 0.38, 0.36, and 0.37, respectively. Lower cut-off values had higher sensitivity but lower specificity for TB pleurisy, while higher cut-off values had higher specificity but lower sensitivity for this condition (Table 2); i.e., for malignant effusions, the 0.4 cut-off value had 90% sensitivity for TB pleurisy, the 8.8 cut-off value had 80% specificity for TB pleurisy, and the 9.5 cut-off value had 85% specificity for TB pleurisy. Of the 101 patients who were diagnosed with TB pleurisy, only 8 (8.8%) had an NLR value of 9.5 or higher. Additionally, 40% of patients with TB pleurisy had an NLR value between 0.4 and 1.4.

## DISCUSSION

In this study, we investigated the cell counts of exudative pleural effusions and these counts' potential contribution to differential diagnosis by calculating the NLR, which can easily be calculated from the pleural fluid cell count and which has been utilized as an inflammatory marker in recent years. The pleural fluid NLR value was significantly lower in TB-related effusion compared to the values in malignant, para-malignant and para-pneumonic effusions. However, this ratio's potential role in differential diagnosis between malignant, para-pneumonic and para-malignant effusions seems to be limited because these conditions present nearly the same NLR values. In any case, in the appropriate clinical context, lower NLR values may be considered as favoring TB pleurisy, while higher values may be used to rule out TB pleurisy. Alternatively, NLR values at least may be used in tailoring the choice of further diagnostic tests, whether invasive or non-invasive.

Many biochemical parameters have been investigated regarding the differential diagnosis of exudative pleural effusion 1-5. Among these parameters, the most frequently studied are the pleural fluid biochemical parameters ADA and CRP as well as parameters, such as the NLR, that easily can be calculated by determining the cell count of the pleural fluid 1,3,5,8,11.

Exudative pleural effusion is present in a variety of pathological conditions in clinical practice and is mostly associated with malignancy and TB 2. The incidence of malignant pleural fluid correlates with age. The most common extrapulmonary form of TB is pleural TB, which specifically occurs in countries with endemic TB 12. According to data from Turkey, the pleural TB rate among extrapulmonary TB cases was 31.1% in 2011. While nearly half of the cases were in individuals between 15 and 35 years of age, 27% were in individuals older than 50 years 13. The differential diagnosis of pleural effusion is difficult, and many laboratory investigations, including invasive procedures, are required. No standard pleural marker has been identified for use in differential diagnosis before moving on to more invasive procedures. For that reason, parameters, such as the NLR, that can easily be calculated and that are widely available have a potential role in the evaluation of exudative pleural effusion.

In a study investigating the role of biochemical parameters in the differential diagnosis of pleural effusion, elevated pleural LDH and low pleural ADA levels were found to be associated with bronchogenic carcinoma; high levels of pleural ADA, with TB; and high levels of both markers, with complicated para-pneumonic effusion or empyema 14. Combined use of ADA and the lymphocyte-neutrophil ratio (LNR) was found to be more useful than use of ADA alone, especially in TB pleural effusion 8,9,15.

The ADA levels in TB pleural effusion are significantly higher compared to those in para-pneumonic, malignant pleural and transudative effusions. In TB pleural effusion, when ADA (≥50 IU/L) is combined with the LNR (≥0.75), the sensitivity and specificity increase 8,15. In a study by Antin et al 9, an LNR value >0.75 in the pleural fluid was a significant indicator of TB pleurisy compared to other exudative effusions, which had lymphocyte predominance.

Previous studies have shown that pleural CRP values are higher in pleural fluid of bacterial origin than in fluid accumulating due to other etiologic causes and significantly higher in exudative fluids compared to transudative fluids 1. Moreover, elevated CRP values in pleural effusion had high sensitivity and specificity for the differential diagnosis of para-pneumonic effusion and other exudative effusions 16. However, the serum NLR value has been shown to be superior to CRP for the differentiation of pulmonary TB and bacterial pneumonia 17. In fact, an NLR value <7 may differentiate pulmonary TB from bacterial pneumonia with a high sensitivity and specificity.

### Strengths and limitations

We compared all possible different exudative pleural effusions and also confirmed our final diagnoses by further invasive diagnostic tests. However, the sample size was relatively small. Moreover, given that calculation of NLR values seemed to be more helpful in TB pleurisy compared to malignant, para-pneumonic and para-malignant effusions, the potential use of this formula may be limited to countries with high TB incidence. Another limitation is that in this retrospective study, a pleural fluid cell count was not performed for many patients, so these patients were excluded from the study because of missing data. An additional limitation of our study was that we only used NLR values; adding other parameters, such as ADA levels, to the evaluation could be more helpful for differential diagnosis.

In conclusion, calculation of the pleural NLR may have a role in the differential diagnosis of TB pleurisy, but not malignant, para-pneumonic or para-malignant effusions. In the appropriate clinical context, lower NLR values may be as favoring TB pleurisy, while higher values may be used to rule out TB pleurisy. Alternatively, NLR values at least may be used in tailoring the choice of further diagnostic tests, whether invasive or non-invasive, especially in countries with a moderate or high TB incidence.

## AUTHOR CONTRIBUTIONS

Akturk UA contributed to the study design, Institutional Review Board application, data collection, data interpretation, literature research, statistical analysis and manuscript composition and revision. She had full access to all of the data in the study and takes responsibility for the integrity of the data and the accuracy of the data analysis. Ernam D contributed to the study design and manuscript composition and revision.

Akbay MO contributed to the Institutional Review Board application, data collection, data interpretation, literature research and manuscript composition. Koçak ND contributed to the study design, data collection, literature research, statistical analysis and manuscript composition and revision. Ogur E contributed to the study design, data collection, data interpretation and literature research. Irmak I contributed to the study design, data collection, data interpretation and literature research.

## Figures and Tables

**Figure 2A:**
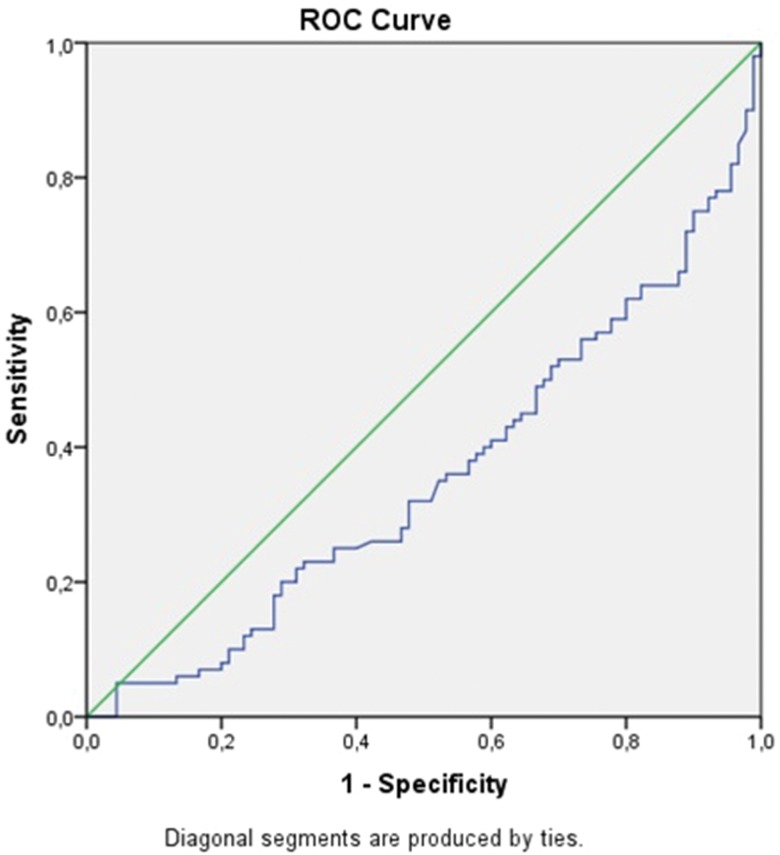
ROC curve for the NLR by TB pleurisy and malignant pleural effusion.

**Figure 2B:**
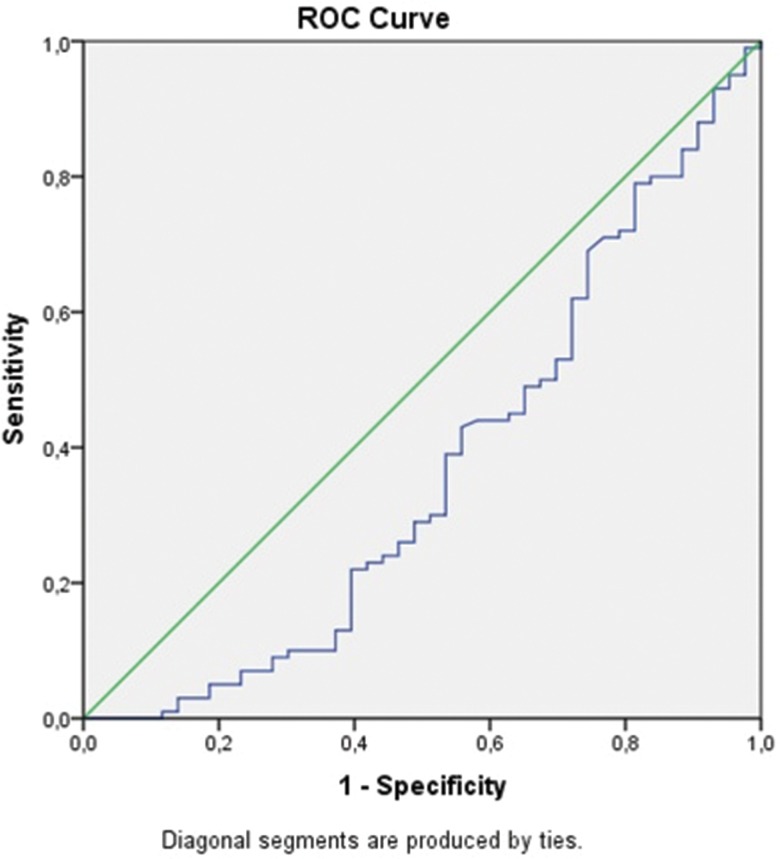
ROC curve for the NLR by TB pleurisy and para-pneumonic pleural effusion.

**Figure 2C:**
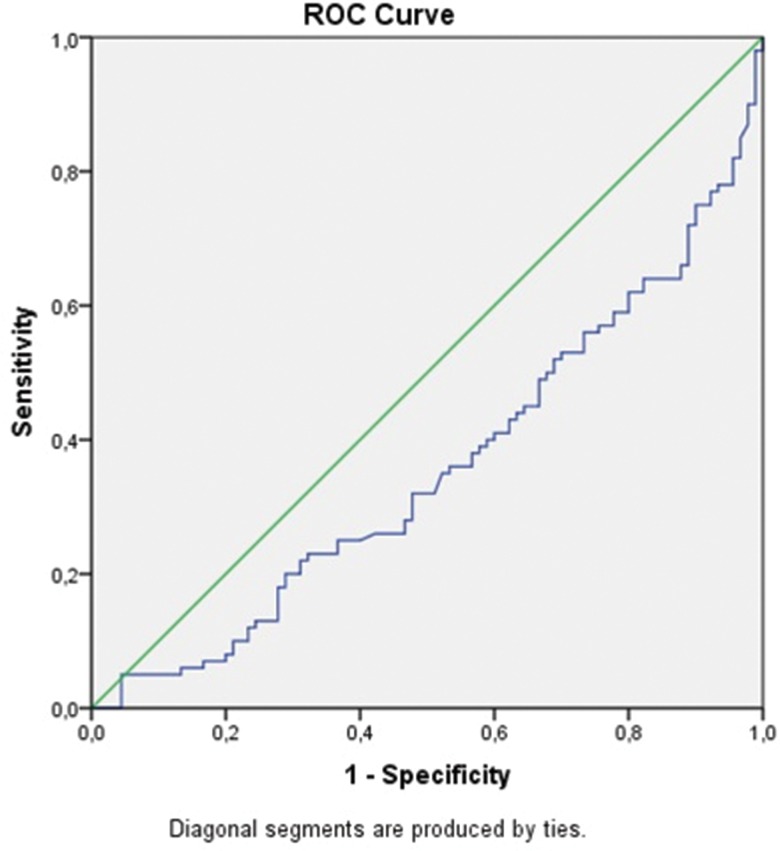
ROC curve for the NLR by TB pleurisy and para-malignant pleural effusion.

**Table 1:** Comparison of pleural fluid NLRs between the exudative pleural disease groups.

Diagnosis (n)	Median (IQR)	*p*-value
Malignant pleural effusion	3.5 (1.6-7.5)	**0.001**
TB pleurisy	2.2 (0.9-5.3)	
Malignant pleural effusion	3.5 (1.6-7.5)	0.48
Para-pneumonic pleural effusion	4.1 (1.8-7.4)	
Malignant pleural effusion	3.5 (1.6-7.5)	0.58
Para-malignant effusion	4.2 (1.0-11.8)	
TB pleurisy	2.2 (0.9-5.3)	**0.001**
Para-pneumonic pleural effusion	4.1 (1.8-7.4)	
TB pleurisy	2.2 (0.9-5.3)	**0.012**
Para-malignant effusion	4.2 (1.0-11.8)	
Para-malignant effusion	4.2 (1.0-11.8)	**0.31**
Para-pneumonic pleural effusion	4.1 (1.8-7.4)	

IQR: Interquartile range

**Table 2:** Cut-off values of the pleural NLR in the differentiation of TB pleurisy from other disease and malignant, para-pneumonic, and para-malignant effusions.

Cut-off value of NLR	Sensitivity %	Specificity %	AUC
TB pleurisy and malignant pleural effusion			
0.4	90.0	2.0	0.38
0.7	80.0	6.0	
1.5	62.0	22.0	
6.0	22.0	70.0	
9.5	10.0	85.0	
TB pleurisy and para-pneumonic effusion			
0.4	90.0	3.0	0.36
0.7	80.0	5.0	
1.2	66.0	14.0	
6.2	20.0	70.0	
12.0	6.0	85.0	
TB pleurisy and para-malignant effusion			
0.4	90.0	7.0	0.37
0.7	80.0	14.0	
1.2	65.0	26.0	
9.2	10.0	70.0	
22.0	3.0	85.0	

TB pleurisy and other disease*
